# Real-World, Single-Center Analysis of Cutaneous Adverse Events with Nemolizumab: Toward Safer and More Effective Use

**DOI:** 10.3390/jcm14248657

**Published:** 2025-12-06

**Authors:** Akiko Sugiyama, Takeshi Nakahara, Kaoru Kojima, Haruko Nishie, Miku Nishimura, Tetsuya Hiramoto

**Affiliations:** 1 Department of Allergology, NHO Fukuoka National Hospital, Fukuoka 811-1394, Japan; 2 Department of Dermatology, NHO Fukuoka National Hospital, Fukuoka 811-1394, Japan; 3 Department of Dermatology, Graduate School of Medical Science, Kyushu University, Fukuoka 812-8582, Japan; 4 Department of Clinical Research, NHO Fukuoka National Hospital, Fukuoka 811-1394, Japan

**Keywords:** atopic dermatitis, nemolizumab, cutaneous adverse events, TARC, risk factors

## Abstract

**Background/Objectives:** Nemolizumab provides rapid and effective relief from pruritus in patients with atopic dermatitis. However, it is frequently associated with cutaneous adverse events, and reliable predictors of their severity have not yet been clearly identified. This study aimed to investigate the relationship between the severity of nemolizumab-associated cutaneous adverse events and patients’ clinical background and to explore baseline factors that may be useful in predicting their severity. **Methods:** We retrospectively analyzed data from 40 patients with atopic dermatitis who received nemolizumab between May 2023 and March 2025. Clinical variables included demographics, prior therapies, phenotype, baseline Eczema Area and Severity Index subscores, serum biomarker levels, and treatment courses. The severity of cutaneous adverse events was classified as mild (<10% body surface area or limited to dryness/desquamation) or moderate-to-severe (≥10% body surface area). **Results:** Cutaneous adverse events occurred in 31 of 40 patients (78%); 13 were moderate-to-severe and 18 were mild. Most events appeared within 16 weeks of treatment initiation. Severity was associated with age, duration of disease, serum Thymus and Activation-Regulated Chemokine (TARC) level, and clinical phenotype. Patients with trunk-dominant phenotypes showed more severe cutaneous adverse events than patients with extremity-dominant or prurigo-type atopic dermatitis. Most cutaneous adverse events resolved within 12 weeks using topical therapy, without requiring treatment discontinuation. **Conclusions:** Baseline characteristics such as age, duration of disease, serum TARC levels, and severity of trunk lesions may be useful in predicting the risk of severe cutaneous adverse events, supporting their potential use in pre-treatment assessment and patient counseling.

## 1. Introduction

Nemolizumab is a humanized monoclonal antibody that targets the interleukin-31 receptor A (IL-31RA), thereby blocking IL-31-mediated pruritus signaling pathways [1,2,3,4]. In Japan, it is approved for the treatment of pruritus associated with atopic dermatitis (AD) in patients aged ≥6 years and for prurigo nodularis in those aged ≥13 years [5]. For AD, the recommended dose is 60 mg every four weeks for patients aged ≥13 years [4,6,7] and 30 mg for those aged 6 to <13 years [8,9]. Nemolizumab provides a rapid and potent antipruritic effect in patients with AD [10,11,12].

Since its approval in Japan in March 2022, nemolizumab has been increasingly used in routine clinical practice. Although clinical trials reported cutaneous adverse events at rates of approximately 10–30% [5,6,8,9,12], real-world observations have revealed a broader range of reactions and a seemingly higher frequency [13,14,15]. Most events responded to adequate topical therapy and improved within two months; however, some patients experienced painful eruptions, alopecia, or widespread lesions that significantly affected their quality of life (QOL). These heterogeneous presentations, together with the potential psychological impact of transient worsening of skin inflammation, highlight the importance of recognizing patients who may be predisposed to more severe reactions. Nevertheless, despite these clinical observations, it remains unclear which baseline factors can reliably predict the severity of cutaneous adverse events. We previously reported that the Eczema Area and Severity Index (EASI) score of the trunk at baseline may serve as a potential predictor [16]. While nemolizumab can often be continued with appropriate topical therapy, even in the presence of cutaneous adverse events, more extensive or severe cutaneous adverse events can negatively impact patient quality of life (QOL) and reduce adherence to treatment. Therefore, identifying patients at higher risk before initiating therapy may help support shared decision-making and long-term treatment planning.

In this study, we aimed to investigate the relationship between baseline patient characteristics and the severity of cutaneous adverse events following nemolizumab initiation.

## 2. Materials and Methods

We retrospectively analyzed the medical records of 40 patients with AD who received systemic treatment with nemolizumab between May 2023 and March 2025, all of whom had completed at least four doses. The study cohort comprised 19 males and 21 females, with a mean age of 14.0 (range, 6–55) years. The following clinical parameters were collected: age, prior treatments before nemolizumab initiation, duration of AD, baseline clinical phenotype, site-specific severity of skin lesions, serum thymus and activation-regulated chemokine (TARC) levels measured by a chemiluminescence immunoassay, total serum immunoglobulin E (IgE), peripheral eosinophil counts, the frequency and timing of cutaneous adverse events, their severity and management, and whether nemolizumab treatment was continued.

In the present study, the severity of cutaneous adverse events was classified based on their potential impact on patient QOL, and patients were accordingly divided into two groups: no/mild cutaneous adverse events and moderate-to-severe cutaneous adverse events. We defined all newly occurring skin manifestations that had not been present before the initiation of nemolizumab, including alopecia and dyshidrotic eczema, as cutaneous adverse events.

The severity of cutaneous adverse events was defined as follows:

**Mild:** Involvement of less than 10% of the body surface area (BSA) or limited to dryness and desquamation.

**Moderate-to-severe:** Involvement of 10% or more of the BSA.

Serum TARC levels measured within one month prior to nemolizumab initiation and total serum IgE levels measured within three months were included in the analysis. The severity of skin lesions was evaluated using the EASI, which assesses erythema, edema/papulation, excoriation, and lichenification across four anatomical regions: head/neck, trunk, upper extremities, and lower extremities.

Patients were classified into four clinical phenotypes at baseline according to the dominant distribution of skin lesions: head/neck-dominant, trunk-dominant, extremity-dominant, and prurigo-type. Patients with trunk- or extremity-dominant AD accompanied by prurigo nodularis lesions were categorized as prurigo-type. This phenotyping approach was restricted to eczema-like reactions and was not designed to generalize to all dermatologic adverse events.

For patients whose duration of disease was not precisely recorded, values were standardized as follows: “≥10 years” was assigned as 10 years, and “≥20 years” as 20 years.

Our institution provides allergy care for both pediatric and adult patients and routinely manages a substantial number of individuals with atopic dermatitis (AD). More than 50 AD patients are seen each month in the Departments of Allergology and Dermatology, most of whom have moderate-to-severe levels of disease. Although many eligible patients have already initiated systemic therapy, approximately 3–4 patients per month newly start treatments such as nemolizumab. These clinical characteristics reflect the typical AD population managed at our center and clarify the clinical context of the cohort included in this study.

This study was conducted retrospectively, and study information was provided to patients using an opt-out approach. The study protocol was approved by the Ethics Committee of the National Hospital Organization Fukuoka National Hospital (Approval No. F6-44; approved on 21 March 2025). As this was a retrospective study and some patients were no longer in active follow-up, it was not feasible to obtain written or verbal informed consent from all participants. Therefore, in addition to verbal consent obtained in routine clinical practice, an opt-out approach approved by the Ethics Committee was implemented for this study.

Statistical analyses were performed using EZR (Easy R; Saitama Medical Center, Jichi Medical University, Japan), a graphical interface for R (The R Foundation for Statistical Computing), available at http://www.jichi.ac.jp/saitama-sct/SaitamaHP.files/statmed.html (version 1.68; accessed on 1 December 2025) [17]. A *p*-value of <0.05 was considered statistically significant. Multivariate analyses were performed, including total or regional EASI score, total IgE level, sex, serum TARC level, and duration of disease. To calculate cutoff values, the Youden index was derived from the sensitivity and specificity curves.

## 3. Results

The incidence of cutaneous adverse events was 31 of 40 patients (77.5%). Cutaneous adverse events were observed in 16 of 19 male patients (84%) and 15 of 21 female patients (71%). Among the 31 patients who developed such events, 13 were classified as moderate-to-severe and 18 as mild. No significant difference in incidence was observed between sexes (Table 1).

The timing of onset for common cutaneous adverse events, including dryness/desquamation, edematous erythema, nummular eczema, and dyshidrotic eczema, was mainly within the first four doses of nemolizumab. Topical corticosteroids were the primary management strategy. For moderate-to-severe cases, the strongest class of topical corticosteroids was used. In some patients, systemic corticosteroids, treatment discontinuation, or changes in systemic therapy were required (Table 2).

In the multivariate regression model incorporating total EASI score, total IgE level, sex, serum TARC level, and duration of disease, serum TARC level remained significantly associated with cutaneous adverse event severity (estimate = 0.00019, SE = 6.38 × 10^−5^, t = 2.966, *p* = 0.01), while the other variables were not significant (Table 3).

### 3.1. Age (Figure 1)

Among patients aged ≤12 years (30 mg dose group), only one developed moderate-to-severe cutaneous adverse events. Events in this age group were milder compared to those in patients aged ≥13 years (*p* = 0.033). When categorized into three age groups (≤12, 13–18, and ≥19 years), a trend toward increased severity with advancing age was observed (*p* = 0.066).

**Figure 1:**
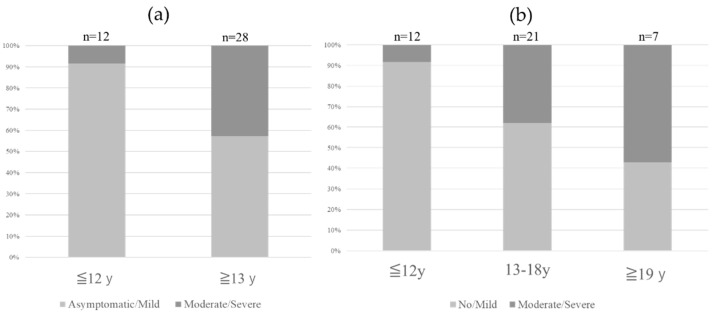
Age and severity of cutaneous adverse events. Cutaneous adverse events in the group aged ≤12 years were significantly milder than those in the patients aged ≥13 years group ((**a**), *p* = 0.033). When classified into three age groups (≤12, 13–18, and ≥19 years), there was a tendency for adverse events to become more severe with increasing age ((**b**), *p* = 0.066).

### 3.2. Clinical Phenotype (Figure 2)

The cohort included 6 head/neck-dominant, 6 trunk-dominant, 19 extremity-dominant, and 9 prurigo-type cases. Fisher’s exact test showed a significant association between clinical phenotype and severity of cutaneous adverse events (*p* = 0.0074). Compared to trunk-dominant cases, extremity-dominant and prurigo-type cases showed significantly milder events (*p* = 0.0028 and *p* = 0.035, respectively).

**Figure 2:**
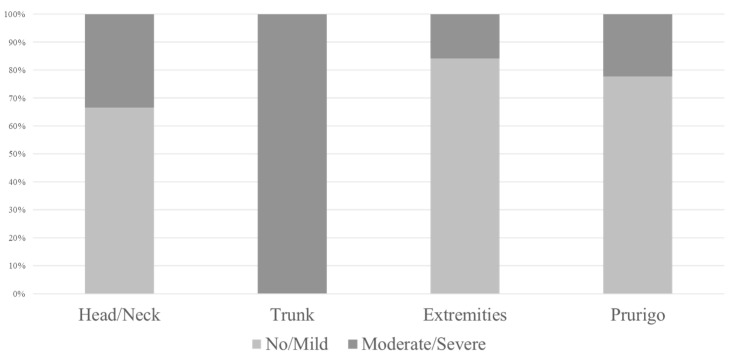
Clinical phenotype and severity of cutaneous adverse events. Compared to trunk-dominant cases, extremity-dominant and prurigo-type cases showed significantly milder events.

### 3.3. EASI Scores of the Trunk (Table 4)

EASI subscores for erythema, edema/papulation, and excoriation in the trunk were significantly higher in the moderate-to-severe group compared to the mild group (*p* < 0.0001, *p* = 0.00067, and *p* = 0.03361, respectively).

**Table 4:** EASI scores of the trunk and severity of cutaneous adverse events.

EASI Score		Trunk			
		Erythema	Edema/Papulation	Excoriation	Lichenification
	*n*	27	27	27	27
No/Mild	Average	1.185185	1.148148	1.055556	0.666667
	SD	0.419435	0.662379	0.763763	0.620174
	*n*	13	13	13	13
Moderate/ Severe	Average	1.884615	1.769231	1.730769	0.769231
	SD	0.219265	0.388125	0.926809	0.438529
*t*-test	*p*	*p* < 0.0001 **	0.00067 **	0.03361 **	0.5514

** Statistically significant (*p* < 0.01).

### 3.4. Duration of Disease (Figure 3)

Longer duration of disease was associated with an increased risk of developing moderate-to-severe cutaneous adverse events (*p* = 0.0372).

**Figure 3:**
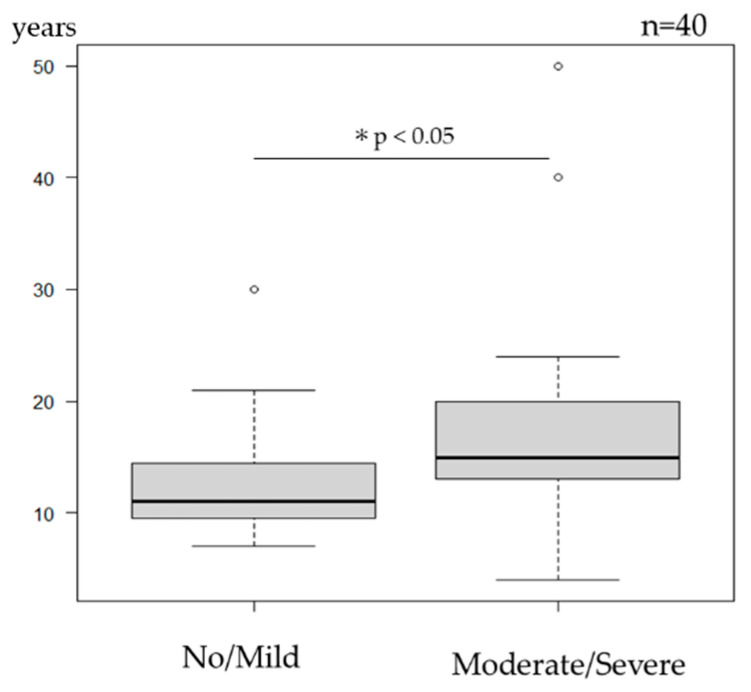
Duration of disease and severity of cutaneous adverse events. The duration of disease was correlated with the severity of cutaneous adverse events (*p* = 0.0372 *). * Statistically significant (*p* < 0.05).

### 3.5. Total Serum IgE (Figure 4)

Among the 22 patients with available IgE data, no significant association was found between total serum IgE and the severity of cutaneous adverse events (*p* = 0.26).

**Figure 4:**
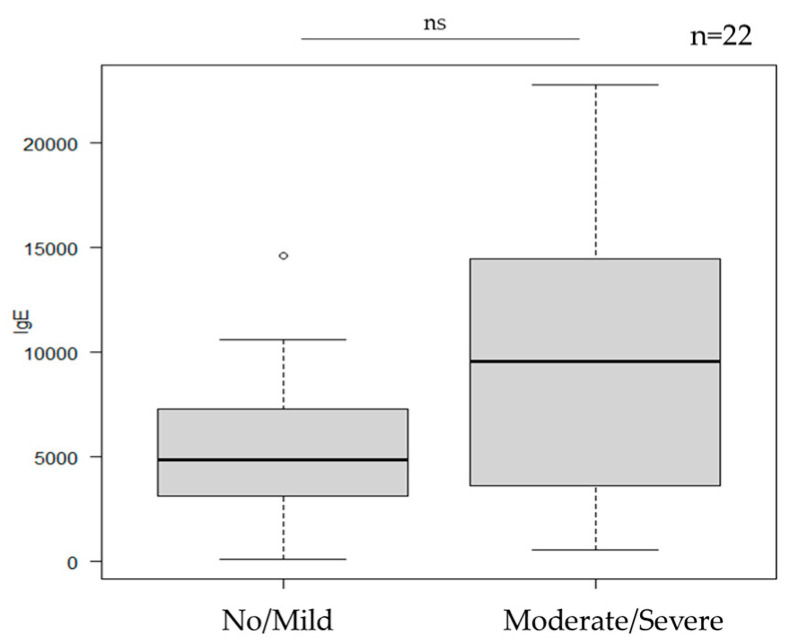
T-IgE and the severity of cutaneous adverse events. No correlation was observed between total IgE levels and the severity of cutaneous adverse events (*p* = 0.26). ns: not significant.

### 3.6. Serum TARC Levels (Figure 5)

Among the 19 patients with available serum TARC data, higher baseline serum TARC levels were significantly associated with moderate-to-severe cutaneous adverse events (*p* = 0.0014). The optimal cutoff value determined by the Youden index was 767 pg/mL (AUC = 0.9359), effectively distinguishing mild from moderate-to-severe reactions.

**Figure 5:**
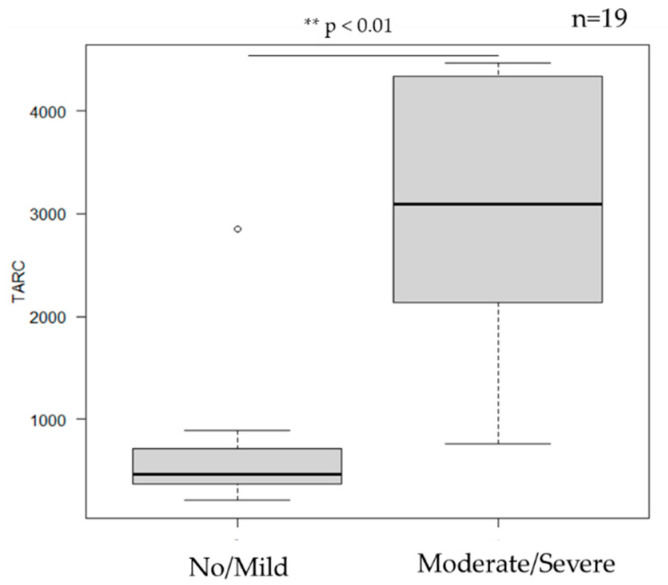
TARC and severity of cutaneous adverse events. TARC levels were significantly associated with cutaneous adverse events (*p* = 0.0014). ** Statistically significant (*p* < 0.01).

### 3.7. Peripheral Eosinophil Counts

Among the 19 patients with available eosinophil data, no significant association was found between eosinophil counts and the severity of cutaneous adverse events (*p* = 0.244; ns). 

## 4. Discussion

Nemolizumab, a monoclonal antibody targeting the IL-31RA, is known for its rapid and potent antipruritic effect, which typically persists for approximately one month following administration [1,2,3,4,5,6,7,8]. For patients with moderate-to-severe AD, in whom pruritus is often the most burdensome symptom, this therapeutic profile may provide meaningful clinical benefit.

In the present study, cutaneous adverse events were observed in approximately 80% of patients, which was higher than the 10–30% incidence reported in previous clinical trials [5,6,8,9,12,15]. This discrepancy might reflect differences in patient demographics. In particular, our cohort included a higher proportion of pediatric and adolescent patients. Additionally, more meticulous documentation of even minor skin changes in routine clinical practice could also have contributed to the increased incidence. The earliest cutaneous adverse events were dryness and desquamation, typically occurring within 4 to 8 weeks of treatment initiation. Subsequently, edematous erythema, nummular eczema, and dyshidrotic eczema were also observed. In some patients, early-onset inflammatory lesions or pain-related symptoms necessitated modification of therapy. During the early phase of clinical use, limited awareness of these reactions sometimes led to premature discontinuation in cases that, in retrospect, might have been managed with appropriate supportive care. These findings underscore the importance of careful monitoring, especially during the first three months of treatment, which appears to represent a crucial period for the detection of cutaneous adverse events. In this study, we did not use the Common Terminology Criteria for Adverse Events (CTCAE) to classify the severity of cutaneous adverse events. The primary reason is that these skin reactions represent adverse events characteristic of nemolizumab and are not directly comparable to those caused by other pharmacologic agents. Moreover, because nemolizumab is positioned as a treatment for atopic dermatitis, we considered it more clinically appropriate to evaluate the severity of these cutaneous reactions within the context of AD management. Therefore, we classified severity based on the extent of skin involvement, using the 10% body surface area threshold commonly applied in the assessment of AD severity.

This study examined not only the frequency of cutaneous adverse events but also baseline factors that may be associated with moderate-to-severe reactions, which are more likely to impair QOL and result in treatment discontinuation. Age appeared to be an important factor: patients aged ≤12 years (receiving 30 mg) tended to experience fewer severe events compared with those aged ≥13 years (receiving 60 mg). This difference may reflect, at least in part, a dose-related effect, although hormonal or immunologic changes during adolescence could also contribute and warrant further investigation.

A longer duration of disease appeared to be linked to an increased risk of moderate-to-severe cutaneous adverse events, which may be related to chronic skin barrier dysfunction and persistent immune dysregulation. Additionally, patients with a trunk-dominant clinical phenotype or higher EASI scores in the trunk region were more likely to develop extensive reactions. This trend contrasts with prurigo nodularis, where lesions commonly arise on the extremities, which are more prone to scratching. Inflammation in less-scratchable areas, such as the trunk, may indicate different patterns of underlying neuroimmune activity.

Among laboratory findings, elevated serum TARC levels appeared to be associated with an increased risk of moderate-to-severe cutaneous adverse events. Based on the ROC analysis, the AUC was 0.9359, and the cut-off value determined by the Youden index was 767 pg/mL, indicating that patients with higher serum TARC levels may be more susceptible to developing severe reactions. A threshold of 767 pg/mL may serve only as a reference; the results suggest that patients with higher serum TARC levels may be more susceptible to developing severe reactions. In contrast, total serum IgE and eosinophil counts showed no significant correlation with the severity of cutaneous adverse events, consistent with previous reports [13]. Although the mechanisms underlying the diverse spectrum of skin reactions remain incompletely understood, several immunologic hypotheses exist. One possibility is that IL-31 blockade induces compensatory upregulation of Th2 cytokines such as IL-4 and IL-13 [18], which could partly explain the development or exacerbation of eczematous lesions, including atopic dermatitis. Alternatively, suppression of the Th2 axis may lead to a relative activation of Th1 and Th17 pathways [19,20], which may in turn account for psoriasiform or pustular eruptions, such as palmoplantar pustulosis. In addition, IL-31 inhibition may modulate TRPV1 activity, thereby affecting nociceptive thresholds. Since TRPV1 is involved not only in itch but also in pain perception, suppression of pruritus could relatively enhance pain sensitivity [21,22], potentially contributing to pain-related symptoms. Furthermore, IL-31 blockade may lead to suppression of STAT3 phosphorylation. Given that STAT3 is known to play a role in regulating the hair cycle [23], its suppression might contribute to alopecia observed in some patients. In our study, alopecia was not pre-existing but newly emerged after the initiation of nemolizumab. These cases were characterized by rapid and substantial hair shedding, partial thinning of the scalp, and the absence of root sheaths on shed hairs—features consistent with telogen effluvium. All cases improved promptly within two months of discontinuing nemolizumab, supporting the interpretation that the alopecia was a nemolizumab-related adverse event.

Clinically, most cutaneous adverse events were manageable with intensified topical corticosteroid therapy, in line with the management algorithm proposed by Mima et al. [13]. However, in cases where symptoms persisted for several months and systemic corticosteroids were required or caused substantial impairment in quality of life, such as alopecia or pain-related disorders, early clinical judgment and modification of the treatment strategy were necessary.

This study may provide insights for clinical practice by exploring baseline characteristics that could be related to the severity of cutaneous adverse events associated with nemolizumab. These factors included age, duration of disease, clinical phenotype, trunk EASI scores, and serum TARC levels. Nonetheless, this study has several limitations. These include a modest and selectively enrolled sample size, incomplete biomarker data, lack of a standardized dosing and washout protocol, single-center design with an ethnically homogeneous population, and the absence of patient-reported outcome measures. In particular, the limited number of cases, especially those with moderate-to-severe adverse events, precluded robust multivariate analysis. As the number of patients was small, the results of the multivariate analyses should be regarded only as reference values. Although serum TARC levels showed a stronger statistical association than other variables, the small sample size suggests that this result should also be interpreted with caution and considered exploratory. Moreover, the washout period for prior therapies was not standardized, making it difficult to fully exclude their influence. Additionally, although alopecia was observed as a potential adverse event, detailed trichoscopic examination was not performed; thus, the absence of objective trichoscopic assessment represents a further limitation in accurately characterizing the nature of this reaction. Further prospective studies involving larger and more diverse populations will be necessary to clarify these preliminary observations.

## 5. Conclusions

Age, duration of disease, trunk EASI scores, and serum TARC levels may serve as possible indicators for identifying patients who could be at increased risk of moderate-to-severe cutaneous adverse events following nemolizumab initiation. Incorporating these factors into pretreatment evaluation might help support risk communication, treatment planning, and long-term adherence, although additional studies are needed for confirmation.

## Figures and Tables

**Table 1:** Clinical summary of cases.

	Severity	
	Mild	Moderate/Severe
**Age**	13 (7–26)	15 (12–55)
**Sex (Male/Female)**	9/9	7/6
**EASI**	15.55 (10.4–31.9)	16.5 (10.8–34.3)
**Prior treatment**	Dupi: 1	Dupi: 1, JAKi: 2
**Number of doses**	2 (1–8)	1 (1–4)
**Dry Desquamation**	9	5
**Nummular Eczema**	2	2
**Edematous Erythema**	4	11
**Dyshidrotic Eczema**	4	2
**Palmoplantar pustulosis**	1	0
**alopecia**	2	0
**Recovery period (months)**	2 (1–16)	2 (1–4)
**Alternative treatment**	Dupi: 1	Dupi: 5, Leb: 2

Dupi: Dupilumab; Leb: Lebrikizumab; JAKi: JAK inhibitor.

**Table 2:** Treatment and outcome.

Severity	Treatment	Continued	Discontinued
Affected Area (% of BSA)			
**Mild** **(<10% of BSA or only dry desquamation)**	Strong TCS	1	
	very strong TCS	9	3 (2 alopecia, 1 nummular eczema)
	strongest TCS	4	
	Difamilast	1	
**Moderate/Severe (** **≥10** **%** **of BSA)**	Strong TCS		
	very strong TCS	3	3
	strongest TCS	3	4
		with oral corticosteroids: 1	with oral corticosteroids: 3
			with oral NSAIDs: 1

BSA: Body Surface Area; TCS: Topical Corticosteroids; NSAIDs: Non-Steroidal Anti-Inflammatory Drugs.

**Table 3:** Multivariable analysis of clinical factors related to moderate-to-severe cutaneous adverse events.

	Estimate	Std. Error	t Value	Pr (>|t|)
Total EASI	0.017781141	0.014955402	1.189	0.2575
Total IgE	0.000005083	0.000018614	0.273	0.7894
sex	−0.246755979	0.161713216	−1.526	0.153
TARC	0.000189302	0.000063834	2.966	0.0118 *
Duration of disease	0.004893328	0.008237808	0.594	0.5635

* Statistically significant (*p* < 0.05).

## Data Availability

The data presented in this study are not publicly available due to patient privacy and ethical restrictions. De-identified data may be shared by the corresponding author upon reasonable request and with approval from the institutional ethics committee.

## Abbreviations

The following abbreviations are used in this manuscript:

AD	Atopic Dermatitis
EASI	Eczema Area and Severity Index
TARC	Thymus and Activation-Regulated Chemokine
QOL	Quality of Life
